# Quantitative determination of selenium in the most common food items sold in Egypt

**DOI:** 10.1186/s42506-020-00044-z

**Published:** 2020-07-08

**Authors:** Fawzya Moatkhef, Hanaa Ismail, Neveen Agamy, Samar Aborhyem

**Affiliations:** 1 Al-Thawra General Hospital, Al-Hadidiya, Yemen; 2 Department of Nutrition, High institute of Public Health, Alexandria University, Alexandria, Egypt

**Keywords:** Selenium, Food samples, Egypt

## Abstract

Particular interest in selenium (Se) was generated as a result of clinical studies showing that balanced Se dietary system is very important for many physiological processes. There is no recent information available on the Se content in Egyptian foods. The present study was conducted to measure Se content in different food groups. A cross-sectional study was designed; a total of 87 food items were randomly purchased from the main markets and hypermarkets in Alexandria governorate, then digested by wet ashing procedure and finally analyzed using Inductively Coupled Plasma-Mass Spectrometry (ICP-MS). The highest mean Se value was obtained in protein-rich food followed by nuts and sweetened products (6.8, 6.2, and 5.89 μg/g respectively) shrimps had the highest value among all studied samples (6.8 μg/g), while the lowest one was in soft cheese (0.0036 μg/g). Selenium content in food groups is strongly correlated with food matrix and composition of food items, soil composition, and fortification process.

## Introduction

The impact of dieting on human health has received such a great care in the last few years with the understanding that unbalanced or deficient diet can cause serious health problems. Three syndromes are associated with Se deficiency: Keshan disease, osteoarthropathy syndrome, and Kashin-Beck disease [1, 2]. Selenium is an extremely important trace element of both nutritional and toxicological interest. Selenium content in food is widely different depending on its concentration in the soil in a given geographical area, the ability of plants to accumulate it, as well as animal feed. Other components such as cultivation, climatic conditions, breeding methods, and methods of preparing food products may also have an effect on Se content in food [3].

Adequate intake of Se in diet reduces risk of heart diseases and low-density lipoprotein (LDL) level in blood, improves immune function, maintains thyroid function, activates synthesis of deoxyribinucleotide, and prevents certain skin infections [4]. Selenium acts as antioxidant, anti-inflammatory, antimutagenic, anticarcinogenic, antiviral, antibacterial, antimycotic, and antiparasitic. A recent study revealed that asthmatic patients have lower level of Se in their blood compared to healthy population [5]. In addition, Se may slow advancement of HIV disease by decreasing oxidative stress and inhibits viral cytotoxic effects [6]. There are at least 30 Se dependent proteins that act as a cofactor in order to convert thyroxine (T4) to bioactive (T3) including glutathione peroxidase and iodothyronine deiodinases enzymes [7].

Epidemiological researches indicated that there is an inverse association between low Se level and prostate, lung, and colorectal cancer. It was found that low selenium intake could accelerate the emergence of cancer cells. There was a small positive association between blood serum Se levels and cancer mortality that highlights the potential application of selenium in cancer prevention and treatment especially among smokers. Also, an association was found between blood Se level and depression, kidney disease, thyroid dysfunction, Alzheimer, liver fibrosis, acute watery diarrhea, and fertility [8, 9].

Excess Se intake may result in many health problems [10–12]. High Se intake in individuals without proved deficit may have bad effects such as hyper-glycaemia, hyper-lipidemia, and depression. Selenium toxicity has been observed after consumption of a large (250 mg) single dose or after multiple doses [13, 14].

Symptoms of high Se intake are strong garlic-like odor, nausea, vomiting, diarrhea, fatigue, skin lesions, decreased cognitive function, weakness, hyperreflexia, pain in the extremities; or paralysis, tremor, muscle spasms, restlessness, confusion, delirium, coma, mucosal damage to the oral cavity, esophagus, and finally death [1, 2].

Selenium compounds are generally very efficiently absorbed by humans; selenium absorption does not appear to be under homeostatic control. For example, absorption of the selenite form of selenium is greater than 80%, whereas that of selenium as selenomethionine or as selenate may be greater than 90%. Therefore, the rate-limiting step determining the overall availability of dietary selenium is not likely to be its absorption but rather its conversion within tissues to its metabolically active forms (e.g., its incorporation into GSHPx or 5-deiodinase) [15].

Selenium is a biologically important element for humans. Different food groups are considered the main sources of Se. It is worthy to know that Se should be taken in specific quantities without increasing or decreasing in order to maintain human health. Unfortunately in many countries all over the world, human food ingredients do not provide sufficient Se [16]. In addition, the data concerning selenium content in food composition tables is often poor and depends on whether analysis is up to date and to what extent natural variability in selenium content is considered. Currently, there is no available data concerning selenium intake among Egyptians [9, 10]. Lack of data about selenium content in Egyptian foods may be one of plausible explanations as selenium is not included in the Egyptian food composition tables; therefore, the present study was conducted to determine the content of Se in different food groups, in order to facilitate the formal decision about dietary selenium intake among Egyptians.

## Materials and methods

### Sample collection

Study included the nine studied food groups***:*** beverages (cola, nescafe, tea, mint, carob, ginger, roselle, caraway***,*** and fenugreek), carbohydrate rich food (toast, shami bread, sweet potato, vino bread, macaroni with whole grain, rice, pasta, potato, local bread***,*** and corn), milk and its product (soft cheese, skimmed milk, processed cheese, fat-free yogurt, semi-hard cheese, roomy cheese, raw milk, full-cream milk, full-cream yogurt***,*** and local yogurt) meat and its products (luncheon meat, kufta, sausage, sussis, bastrama, veal meat, cow’s kidney, beef, sheep meat, and cow’s liver), fruits and vegetables (beet, cucumber, apple, garlic, lemon, tomato, onion, orange, carrot***,*** and banana), protein***-***rich food (beans, lentils, egg, chicken, mackerel, mullet, tilapia, duck, crab***,*** and shrimps), nuts (cashew, seed sunflower, watermelon seed, peanuts, pistachio, almond, hazelnut, walnut, and sesame seeds), fats (industrial butter, morta, industrial margarine, animal fat, coconut oil, sunflower oil, corn oil, olive oil, local margarine, and local butter)***,*** and sweetened products(gelatine, tea biscuits, jam, galaxy chocolate, raw chocolate, chocolate biscuits, sweet tahini, black honey***,*** and bee honey). Nine food items were taken from each of the beverages, sweetened products***,*** and nuts, while 10 food items were taken from each of the remaining groups giving the total of 87. Each food item was purchased four times from four different markets in Alexandria governorate giving a total of 348 samples. Pooling was done for each food item (four replicates) by mixing them well together to obtain random and representative sample for each. Samples were collected during the period of January***–***March 2018.

### Sample storage

Samples were collected and protected from contamination or Se loss during analysis. Samples as meat, fish, and seafood were stored at − 20 °C till analysis. Yogurt, fruit vegetable, and cheese were analyzed once purchased, while in case of other food items as oil, nuts, cereal, and beverages they were stored at room temperature 27 °C until analysis.

### Sample preparation

Solid samples (cereal, legume, and nuts) were grained by grinder, while liquid samples were analyzed directly. Fresh milk and dairy products were collected in a special container to avoid contamination. Fats and connective tissue were discarded from meat and poultry, while in case of seafood only edible parts of tissue were analyzed; head, skin, viscera, scales, and tail were removed. Edible portions of vegetables and fruits were homogenized well in a porcelain mortar to obtain homogeneous samples [17].

### Methods

All samples were prepared for selenium analysis by wet ashing procedure [18]. Sample preparation by wet ashing was categorized according to methods of digestion. Food samples were divided into the following:

Beverages: 100 mg of each sample was added to 10 ml of HNO_3_ (65%), heated on hotplate at 130 °C till dryness. After cooling, 5 ml of H_2_O_2_ (40%) was added and reheated at 130 °C again till dryness. Samples were diluted with 20-ml distilled water and filtered twice through Whatman filter paper grade II to glass bottles. Milk samples: two steps were operated. First step, 2.5 ml of milk samples was added to 4 ml of nitric acid (65%), and 4 ml of hydrogen peroxide (40%) and 4 ml of distilled water were added, then heated at 200 °C till dryness. Second step, addition of 1.4 ml of HCl, then heated at 100 °C, after cooling; 2 ml of H_2_SO_4_ was added and heated at 50 °C then increase temperature to 130 °C till dryness. Samples were diluted and filtrated. Concerning dairy products: 1 g was weighted then added 10 ml of concentrated nitric acid (65%), heated at 100 °C. Then 10 ml of hydrogen peroxide (40%) was added and heated again at120 °C; finally, 2 ml of HCl was added and heated at 200 °C till dryness.

Meat and seafood:1 g of each sample type was weighted and 15 ml HNO3 (65%) was added then heated at 200 °C. After cooling, 15 ml of H_2_O_2_ was added, and then heated at 150 °C. While in case of cereal, legume, and nuts samples, 1 g was added to 20ml nitric acid (65%), 20 ml of hydrogen peroxide, and 20-ml distilled water, and then heated at 170 °C till dryness. Vegetables and fruits: 1 g of sample was added to 10 ml of the HNO_3_ (65%). Samples were kept overnight at the room temperature, then heated at 100 °C. After cooling, 5 ml of HCl was added and heated till dryness. Sweetened products and liquid fat samples: 5 ml of sample was added to 5.0 ml of HNO_3_ and heated at 100 °C till dryness. All the wet-digested samples were ready to determine Se content by Inductively Coupled Plasma-Mass Spectrometry, USA. (Varian 720-ES) [17].

### ICP-MS determination procedure

#### Instrumentation

ICP-MS measurements were performed using a VG Plasma Qua Ex-Cell (Thermo, Courtaboeuf, France). Sample solutions were pumped by a peristaltic pump from tubes arranged on a CETAC Varian 720-ES Inductively Coupled Plasma-Mass-Spectrometry USA (CETAC, Omaha, NE) [2].

#### Optimization

The isotope 78Se, 82Se was selected as analytical masses in ICP-MS instrumental parameters.

#### Operating conditions

Nebulizer: concentric type pumped at 0.9 ml/min. Spray chamber: Scott-type double-pass water cooled. ICP-MS standard mode for Se elements, several specific isotopes were considered and monitored according to the sensitivity of the element and/or possible isobaric and polyatomic interferences. Torch position, ion lenses, and gas output were optimized daily with the tuning solution (1 g/l) to carry out a short-term stability test on the instrument, to maximize ion signals, and to minimize interference effects from polyatomic ions and doubly charged ions. In all experiments, a relative standard deviation (RSD) of 3% was achieved. To obtain precise and accurate results, element signals were monitored by real-time display (RTD), which showed the constant sensitivity over time for the selected masses and the ratios of masses with three readings, calculated for each sample [2].

#### ICP-MS analysis

The diluted solution was filtered through a plastic tube designed for the autosampler of Agilent 4500 Series ICP-MS (Agilent, Palo Alto, CA, USA) that was used for the analysis of selenium content. The analysis conditions were as follows: RF power 1200 W, plasma gas (Ar) 16.0 ml/min, aux. gas (Ar) 1.0 ml/min, carrier gas (Ar) 1.14 ml/min, Barbington-type nebulizer, glass spray chamber, sampling depth 8.2 mm, Ni/Ni sampling cone/skimmer cone, and mass for selenium was m/z 82. Selenium concentrations of the samples were calculated from the regression line (*r*^2^ = 0.999) obtained using selenium standard solution (Wako Pure Chemical Industries, Osaka, Japan) [19].

The method was validated by analysis of selenium content of NIST Standard Reference Material 8436 durum wheat flour obtained from National Institute of Standards and Technology (Boulder, CO, USA), and the recovery tests were performed on some food groups spiked with selenium standard and the recovery value ranged (0.002–0.05 μg/g) [19].

### Statistical analysis of the data

Data were fed to the computer and analyzed using the IBM SPSS software package version 20.0. (Armonk, NY: IBM Corp.). Qualitative data were described using number and percent. The Kolmogorov-Smirnov test was used to verify the normality of distribution. Quantitative data were described using range (minimum and maximum), mean, standard deviation, and median. Significance of the obtained results was judged at the 5% level. The used tests were Kruskal–Wallis test for abnormally distributed quantitative variables, to compare between more than two studied groups and post hoc (Dunn’s multiple comparisons test) for pairwise comparisons [19, 20].

## Results and discussion

Despite the fact that fruits contain low levels of Se because of its high water and low protein content [21], the present study revealed that the value of Se in banana, orange, and apple was 2.3, 1.3, and 0.84 μg/g respectively as shown in Table 1. This may be attributed to agricultural activities that may influence Se levels in foodstuffs, where Se has been added to fertilizers in some areas of the world in order to increase Se levels in cultivated plants and indirectly improve Se status in humans. In comparison to other studies, Se content in the present study was higher than that given for fruits grown or purchased in eastern Croatia [22]. Meanwhile, Klapec et al. [22] results came in agreement with current results in descending order of selenium in fruits where Se values in banana, orange, and apple were 0.203, 0.076, and 0.088 μg/g, respectively.

**Table 1:** Selenium content in various foods (μg/g)

Beverages		Mean ± S.D	Nuts		Mean ± S.D	Sweetened products		Mean ± S.D
Sample	(μg/g)		Sample	(μg/g)		Sample	(μg/g)	
Cola drink	0.002 ± 0.001	0.69 ± 1.44	Cashew	0.028 ± 0.013	2.29^a^ ± 1.94	Gelatin	0.001 ± 0.001	1.56^a^ ± 1.92
Instant coffee	0.005 ± 0.001		Seed sunflower	0.51 ± 0.01		Tea biscuits	0.26 ± 0.18	
Tea	0.0064 ± 0.001		Watermelon seeds	0.96 ± 0.21		Jam	0.35 ± 0.22	
Mint	0.014 ± 0.005		Peanuts	2.14 ± 0.51		Galaxy	0.62 ± 0.47	
Carob	0.018 ± 0.010		Pistachio	2.24 ± 0.68		Raw chocolate	0.94 ± 0.17	
Ginger	0.028 ± 0.013		Almonds	3.25 ± 0.29		Chocolate biscuits	1.61 ± 0.59	
Roselle	0.35 ± 0.12		Hazelnut	3.56 ± 0.33		Sweet tahini	2.36 ± 0.83	
Caraway	2.20 ± 0.26		Walnuts	4.22 ± 0.10		Black honey	3.10 ± 0.15	
Fenugreek	4.30 ± 0.70		Sesame seeds	5.89 ± 0.84		Bee honey	6.20 ± 0.62	
Carbohydrate-rich food		Mean ± S.D	Protein-rich foods		Mean ± S.D	Fat		Mean ± S.D
Sample	(μg/g)		Sample	(μg/g)		Sample	(μg/g)	
Toast	0.014 ± 0.011	0.36^b^ ± 0.64	Bean	0.26 ± 0.047	3.07^ad^ ± 2.17	Industrial butter	0.0033 ± 0.002	1.74^a^ ± 1.86
Shami bread	0.023 ± 0.021		Lentils	0.42 ± 0.326		Morta	0.026 ± 0.038	
Sweet potato	0.028 ± 0.003		Eggs	1.42 ± 0.556		Industrial margarine	0.041 ± 0.036	
Vino bread	0.045 ± 0.017		Poultry	2.2 ± 0.588		Animal fat	0.12 ± 0.10	
Macaroni with whole grain	0.11 ± 0.098		Mackerel	2.4 ± 0.448		Coconut oil	0.26 ± 0.12	
Rice	0.15 ± 0.03		Mullet	3.9 ± 0.826		Sunflower oil	1.8 ± 0.04	
Pasta	0.16 ± 0.04		Tilapia	3.30 ± 0.394		Corn oil	3.2 ± 0.61	
Potatoes	0.48 ± 0.41		Ducks	4.2 ± 0.471		Olive oil	3.4 ± 0.69	
Local bread	0.48 ± 0.41		Crab	5.8 ± 0.850		Local margarine	4.1 ± 0.85	
Corn	2.1 ± 0.46		Shrimp	6.8 ± 0.035		Local butter	4.4 ± 0.14	
Vegetables and fruits		Mean ± S.D	Meat and its products		Mean ± S.D	Milk and dairy products		Mean ± S.D
Sample	(μg/g)		Sample	(μg/g)		Sample	(μg/g)	
Beet	0.024 ± 0.028	1.06 ± 0.68	Luncheon meat	0.001 ± 0.001	1.03^e^ ± 1.22	Soft cheese	0.0036 ± 0.002	0.82^e^ ± 0.93
Cucumber	0.032 ± 0.019		Kufta	0.0022 ± 0.002		Skimmed milk	0.023 ± 0.015	
Apple	0.84 ± 0.021		Sausage	0.0084 ± 0.013		Cheese triangular	0.025 ± 0.022	
Garlic	0.98 ± 0.357		Sussis (sausage)	0.023 ± 0.020		Free-fat yogurt	0.096 ± 0.006	
Lemon	1.1 ± 0.80		Bastrama	0.52 ± 0.21		Semi-hard cheese	0.47 ± 0.08	
Tomatoes	1.1 ± 0.40		Veal meat	0.64 ± 0.56		Rumi cheese	0.68 ± 0.18	
Onions	1.3 ± 0.24		Cow’s kidney	1.48 ± 0.70		Raw milk	0.94 ± 0.05	
Orange	1.3 ± 0.32		Beef	1.8 ± 0.07		Full-cream milk	1.25 ± 0.13	
Carrots	1.6 ± 0.28		Sheep meat	2.2 ± 0.38		Full-cream yogurt	2.12 ± 0.36	
Banana	2.3 ± 0.20		Cow’s liver	3.62 ± 0.71		Local yogurt	2.63 ± 0.31	

^a^Significant with drinks group

^b^Significant with nuts group

^c^Significant with sweetened group

^d^Significant with carbohydrate group

^e^Significant with protein-rich foods group

There is a remarkable increase in Se value in Egyptian fenugreek (4.3 μg/g) (Table 1). This value is not in agreement with other researchers. Askar and Bielig [17] stated that Se content of Egyptian fenugreek was 0.29  μg/g; Karadas [23] stated that content of Se in fenugreek was zero, and Al-Ahmary [24] stated that Se content in Saudi Arabia’s fenugreek seeds was 0.022 μg/g. This may be attributed to enrichment of Egyptian soil with minerals [25].

Regarding selenium content in nuts, it was found that sesame seeds had the highest Se value 5.89 μg/g as presented in Table 1, and this finding is contradicted with Askar and Bielig [17] who stated that, sesame seeds revealed much lower Se content 0.38 μg/g, that may be due to *Askar* study was done in 1983, and also due to more sophisticated and advanced instrument was used in the present study.

Lemire et al. [26] showed that Brazilian walnuts had higher Se content than that detected in the present study 4.22 μg/g. This confirms the theory of Brazilian walnuts are rich in Se content. These differences may be due to geographical, climatologic differences which possibly reflect differences in Se concentration in soil all over the world [27, 28].

Data concerning bee and black honey were 6.2 and 3.1 μg/g respectively as shown in Table 1. These findings are higher than Se value in the study of Costa-Silva et al. [29] who stated that Se content of Portuguese unifloral honeys were ranging from < 0.01 to 0.11 mg/g. This difference may be due to the influence of the geographical and botanical origin of the honeys. Prevalence of the different melliferous flowers and trees harvested by bees is influenced by the soil composition in addition to the climatic conditions and difference in bees species.

Concerning Se content in Egyptian rice, corn, and local bread, it was found that Se values were 0.15, 2.1, and 0.48 μg/g respectively as presented in Table 1. These values were higher than those obtained by Choi et al. [19] who found that Se values in Korean rice, corn, and loaf bread were 0.050, < 0.001, and 0.216 μg/g, successively. Meanwhile, Se value in macaroni with whole grain 0.11 μg/g, versus buck wheat noodles in Choi et al. [19] who demonstrated similar 0.110 μg/g Se value with the present study.

Results concerning Se content in rice was 0.15 μg/g, which was higher than results in Saudi Arabia 0.072 μg/g [24], in Brazil 0.13 μg/g [26], as well as in Thailand 0.05 μg/g [30]. On the other hand, it was lower than that recorded in a Greek study 0.191 μg/g. This may be due to the difference of selenium content in soils from one country to another one [31, 32].

Table 2 shows that protein-rich food contained the highest mean Se 3.07 μg/g ± 2.17 compared to the rest eight groups followed by nuts 2.29 μg/g ± 1.94 then fat group 1.74 μg/g ± 1.86, while food rich in carbohydrate had the lowest mean of Se 0.36 μg/g ± 0.64. There was a significant difference in Se content among the nine studied groups (*P* < 0.005).

**Table 2:** Comparison between the nine studied groups according to selenium content (μg/g)

Groups	*N*	Selenium content (μg/g)			H	*p*
		Min.–Max.	Mean ± SD.	Median		
Drinks	10	0.0–4.30	0.69 ± 1.44	0.02	21.808^*^	0.005^*^
Nuts	10	0.03–5.89	2.29^a^ ± 1.94	2.19		
Sweetened products	10	0.0–6.20	1.56^a^ ± 1.92	0.78		
Carbohydrate	10	0.01–2.10	0.36^b^ ± 0.64	0.13		
Protein-rich foods	10	0.26–6.80	3.07^ad^ ± 2.17	2.85		
Fat	10	0.0–4.40	1.74^a^ ± 1.86	1.03		
Vegetables and fruits	10	0.02–2.30	1.06 ± 0.68	1.10		
Meat	10	0.0–3.62	1.03^e^ ± 1.22	0.58		
Milk and dairy products	10	0.0–2.63	0.82^e^ ± 0.93	0.58		

*H* Kruskal–Wallis test

*p p* value for comparing between the nine groups

*Statistically significant at *p* ≤ 0.05

^a^Significant with drinks group

^b^Significant with nuts group

^c^Significant with sweetened group

^d^Significant with carbohydrate group

^e^Significant with protein-rich foods group

Selenium content in fish is highly variable and depends not only on fish species, but also on fish habitat and season (Table 1) [33]. This statement came in line with the present study, were mullet and tilapia had the highest Se content among other food items (3.9 and 3.30 μg/g respectively) as shown in Table 1. Se content of Mediterranean sea-fish in Alexandria had high levels compared with the fish from tropical Brazilian coast. Seixas et al. [34] who reported that high Se content was found in both carnivorous and non-carnivorous fish (1.63 and 1.08 μg/g respectively). Se content in fish is high and that may be due to high protein content in fish. Fish can be in some cases a poor source of available Se, due to its high content of mercury (Hg) and other heavy metals, which bind to Se forming insoluble inorganic complexes [35]. Choi et al. [19] in Korea revealed lower Se contents in shellfish and their products ranging from 0.152 to 0.788 μg/g, when compared with the present results. Seixas et al. [34] stated that Se content in shrimp was 1.08 μg/g, and it is considered lower than the present results (6.8 μg/g) that may be due to difference in fish habitat and season [28].

Foods of animal origin are assumed to contain high level of Se because selenium is an essential element for the growth of animals [22]. This statement is in agreement with the present results where Se value in eggs was 1.42 μg/g (Table 1). Fortunately, the present study exhibited higher Se value than the study of Yaroshenko et al. [36] in eggs, which was 0.194 μg/g. Choi et al. [19] in Korea revealed that Se content in eggs was 0.267 μg/g; Lemire et al. [26] also found that eggs (yolks and white) had Se values 0.56 and 0.21 μg/g, respectively. Gao et al. [37] reported that Se content in eggs in China was 0.152 μg/g. Elevated results concerning eggs may be as a result of enriched animal feed in Egypt [38].

Selenium content in various fat revealed that Se in olive oil, corn oil, local margarine, and local butter were 3.4, 3.2, 4.1, and 4.4 μg/g respectively. These findings were much higher than that in Al-Ahmary [24] in Saudi Arabia, who found that Se values in olive oil, corn oil, margarine, and butter were 0.002, 0.007, 0.002, and 0.005 μg/g, respectively. Local products were rich in selenium; possibly enriched animal feed and raw materials of olive oil, corn oil, and local margarine are rich with minerals.

Results of the present study as regards garlic and onion demonstrated that Se content was 0.98 and 1.3 μg/g respectively. These results highlighted that Allium genus (onion, garlic) tends to accumulate Se probably because of their greater fraction of sulfur-containing amino acids [21], where adequate analogs of these can be formed by substitution of sulfur with Se, resulting in high Se levels. These results were higher than those reported by Askar and Bielig [17] in Egypt who found that Se values in garlic and onion were 0.52 and 0.02 μg/g respectively. Choi et al. [19] in Korea reported that, onions and garlic had 0.052 and 0.021 μg/g, respectively. Lemire et al. [26] in Brazil revealed lower Se values in garlic and onion, 0.08 and 0.07 μg/g, respectively. It is known that garlic and onion tend to have higher Se concentrations.

Results concerning Se content in beef meat were 1.8 μg/g; this finding highlighted that Egyptian meat beef is a good source of Se, due to its high protein and mineral contents. Data of the present study was higher than values reported in Yu et al's study [39] about Se geochemical distribution in the environment and predicted human daily dietary intake which had reported higher soil concentrations of Se. Furthermore, Choi et al. [19] reported that, meats had a range of 0.043–0.324 μg/g. These differences in Se concentration of meat products possibly reflect differences in the Se concentration of the animals feed, where selenium is supplied in the animal diet either in natural organic form (mainly selenomethionine) or in the inorganic form (sodium selenite or selenate). Moreover, difference of Se concentrations in soil worldwide plays important role as well [33, 40].

Selenium values in raw milk, full cream milk, as well as skimmed milk were 0.94, 1.25, and 0.023 μg/g respectively. These results are not expected because it is known that Se concentration in milk is negatively correlated with its fat content. These findings are not in accordance with what was found in other studies [14, 26, 30]. Selenium ratio in milk may be lower than meat and egg due to the fact that milk contains less protein than meat and egg.

Results concerning local yogurt and full-cream yogurt were 2.63 and 2.12 μg/g respectively, while in free-fat yogurt, *Se* value was 0.096 μg/g. These findings are not in agreement with literature which highlighted that Se concentration in milk is negatively correlated with its fat content [26]. They were also higher than what found in other studies; Pappa et al. [31] (2006) in Greek who stated reported that Se content was 0.02 μg in full- or low-fat yoghurt compared to 0.03 μg/g in free fat one [31]. Choi et al. [19] in Korea reported that yoghurt had 0.011 μg/g of Se. That may be due to different methods in preparing raw materials during yoghurt processing and also may be due to the presence of powdered milk in our yoghurt products.

On comparing the significance between the nine studied food groups shown in Table 3, we found that there was a significant difference between the majority of groups (*p* < 0.05), while there was a strong significant difference between the following pairs: drinks versus protein-rich foods and carbohydrate-rich foods versus protein-rich foods as *p* < 0.001.

**Table 3:** Significance and non-significance of differences between nine studied groups according to selenium content

Groups	Beverages	Nuts	Sweetened product	Carbohydrate	Protein-rich foods	Fat	Vegetables and fruits	Meat	Milk and milk products
Beverages		0.003^*^	0.048^*^	0.697	< 0.001^*^	0.044^*^	0.055	0.281	0.260
Nuts			0.338	0.011^*^	0.475	0.353	0.306	0.063	0.070
Sweetened				0.111	0.094	0.976	0.949	0.366	0.392
Carbohydrate					0.001^*^	0.105	0.127	0.491	0.462
Protein-rich foods						0.100	0.082	0.010^*^	0.011^*^
Fat							0.925	0.351	0.376
Vegetables and fruits								0.402	0.428
Meat									0.962
Milk and dairy products									

The *p* value for Dunn’s multiple comparison test comparing between each two groups

*Statistically significant at *p* ≤ 0.05

## Conclusions and recommendations

Protein rich foods had the highest sources of Se in Egyptian products, followed by nuts. On the other hand, soft-drinks and milk products represented the lowest sources of Se*.* Generally, the richest sources of Se in the Egyptian diet were bee honey, fenugreek, sesame, local margarine and butter, ducks, fish, and olive oil. Meanwhile, some foods contained lower proportion of Se but consumed in large quantities such as carbohydrate, vegetables, and fruits, as well as milk and milk products. There are no concerns about the expectation of Se deficiency among Egyptians since no estimation of dietary reference intake was performed. The researchers recommend further study to cover the other unstudied Egyptian food samples.

## Data Availability

The data are available from the corresponding author on reasonable request.
